# RWE Submission for European Regulators and Payers: Challenges, Uncertainties, and Opportunities

**DOI:** 10.1007/s43441-026-00958-4

**Published:** 2026-03-26

**Authors:** Lockwood Taylor, Blythe Adamson

**Affiliations:** Flatiron Health, 233 Spring Street, New York, NY 10013 USA

**Keywords:** Real world evidence (RWE), Joint clinical assessment (JCA), Evidence generation, Europe, Health technology assessment (HTA), European Medicines Agency (EMA)

## Abstract

The new European Union Joint Clinical Assessment (JCA) process, effective January 2025, requires pharmaceutical companies to submit evidence within 100 days of notification. Key challenges include uncertainty about PICO (Population, Intervention, Comparator, Outcomes) parameters prior to PICO notification, limited timeframe for evidence generation, and need to reconcile differences between regulatory versus health technology assessment (HTA) evidence standards. Uncertainties involve predicting PICOs accurately and determining real-world evidence’s (RWE) appropriate role. However, opportunities exist for integrated evidence planning across clinical development, regulatory, and health economics and outcomes research (HEOR) teams, leveraging real-world data to inform or address PICOs as well as support regulatory evidence requirements. Early alignment between key functional teams, combined with proactive RWE use, is essential for generating fit-for-purpose evidence that satisfies the dual scrutiny of regulatory approval and HTA relative effectiveness assessment.

## Introduction

As of January 2025, the European Union’s (EU) Joint Clinical Assessment (JCA) process requires Marketing Authorization Holders (MAHs) to rapidly integrate regulatory and health technology assessment (HTA) evidence generation within the framework established by Regulation (EU) 2021/2282 [1]. Regulation (EU) 2021/2282 established the JCA to address longstanding concerns regarding duplication of clinical assessments across Member States, variability in methodological standards, and delays in patient access arising from sequential national HTA reviews. Participation in JCA is mandatory for eligible centrally authorized medicines, beginning with oncology and advanced therapy medicinal products. The JCA focuses exclusively on the joint assessment of relative clinical effectiveness and safety; economic evaluation, pricing, and reimbursement decisions remain under national authorities [2]. The resulting JCA report informs, but does not replace, national HTA appraisals, which may incorporate local economic analyses and contextual considerations.

The JCA process runs in parallel with European Medicines Agency (EMA) review. After EMA notification of an upcoming marketing authorization application, an HTA assessor and co-assessor are appointed and Population, Intervention, Comparator, Outcomes (PICOs) are harmonized across Member States. Once the finalized PICO list is approved, the MAH has 100 days to prepare and submit the JCA dossier while the EMA application remains under review. Initial products assessed in this new review pathway include tovorafenib (pediatric low-grade glioma) and an autologous tumor-infiltrating lymphocyte therapy (melanoma). In 2025, thirteen products completed or were undergoing JCA evaluation [3]. The compressed timeline, combined with the possibility of numerous and heterogeneous PICOs, makes the use of real-world evidence (RWE) an important consideration. While the new JCA process introduces several challenges and uncertainties, it also offers important opportunities for MAHs to leverage RWE effectively for both regulatory and HTA submissions.

## Challenges

### Heterogeneous PICOs

The new JCA process presents unique challenges for MAHs. Perhaps the most significant challenge is that PICOs are not disclosed until after the MAH submits its EMA application, leaving the required evidence undefined in advance of submission [4]. As PICOs may vary across Member States based on different standards of care and patient populations within each country, evidence needs may be highly heterogeneous. With only 100 days to prepare the JCA dossier, the generation of de novo evidence is often impractical, increasing reliance on pre-existing, accessible real-world data (RWD) platforms and pre-developed analytical protocols. As a result, proactive identification and vetting of data sources may become a potential bottleneck. Although MAHs are strongly discouraged from leaving PICOs unaddressed, published evidence may suffice for some, but not all, PICOs. However, in rare disease areas such as oncology, existing literature may be scant, and a MAH must generate new evidence. Variations in national standards of care can further complicate efforts to produce representative evidence across Member States.

### Divergent Regulatory and HTA Evidence Needs

Regulatory and HTA evaluations often differ in evidentiary focus. Regulatory evidence primarily emphasises randomized controlled trials to ensure internal validity and to support the assessment of a product’s benefit-risk balance for marketing authorization. By contrast, HTA evidence often draws upon indirect treatment comparisons, RWD, and network meta-analyses to evaluate relative clinical effectiveness and cost-effectiveness, informing reimbursement and pricing decisions [5, 6]. These distinct evidentiary needs highlight the importance of understanding both EMA and JCA/HTA body guidance and expectations in defining relevant comparators, data sources, and analytical approaches [7, 8]. Increasingly, methodological approaches such as external comparator arms for single-arm trials may help bridge requirements by generating evidence that is both robust and policy-relevant. However, even with the same methodological approach, regulators and HTAs often differ on endpoints (e.g., progression-free survival versus overall survival in oncology) and statistical methods for comparisons of RWE to clinical trials, requiring careful consideration of data sources, data maturity, and interpretation.

## Uncertainties

### PICO Forecasting

PICO forecasting is an important strategic decision under the JCA model. Modeling and data-driven scenario planning may substantially reduce uncertainty. MAHs face strategic tradeoffs between designing broad evidence packages covering all potential populations, comparators, and outcomes, or targeted approaches focused on major markets. In practice, PICO forecasting may be approached by identifying plausible comparators by Member State based on current standards of care and guidelines, then scoring candidate PICOs by likelihood of selection and market importance. For each high-priority PICO, MAHs may benefit from assessing whether pre-existing RWD sources can support the required population, comparator, and outcomes within the JCA timeline, and flag PICOs that would require de novo evidence generation. This structured “PICO-forecast matrix” can guide early investment in data access and analytic templates, reducing the risk of late-stage evidence gaps once final PICOs are issued. RWE may be used to anticipate likely PICOs and inform comparator selection, though uncertainty remains around its acceptability in providing acceptable supplementary evidence for safety and effectiveness, particularly with single-arm trials.

### Evidence Transportability

Another uncertainty surrounds the feasibility and acceptability of transportability analyses. We recommend that MAHs pre-specify a transportability strategy at the time of evidence planning, including (1) the target PICOs, (2) a priori identification of plausible effect modifiers (e.g., disease severity, biomarker prevalence, prior lines of therapy, performance status), and (3) planned sensitivity analyses to assess robustness to residual confounding and violations of key assumptions. For example, when using US RWD to contextualize a single-arm oncology trial for an EU PICO where subsequent therapy access differs, MAHs may benefit from documenting differences in care pathways (e.g., sequencing, reimbursement-driven availability of later-line therapies) and assessing whether these contextual differences plausibly modify treatment effects. Sensitivity analyses (e.g., re-weighting under alternative modifier sets or bounding analyses for unmeasured modifiers) should be pre-specified to evaluate the stability of conclusions. Transparent documentation of these assumptions and contextual differences may increase HTA confidence in transported estimates and clarify when transportability is infeasible versus decision-relevant.

While transportability analyses can theoretically address evidence gaps in specific PICOs by leveraging high-quality evidence from alternative populations (including non-EU sources), the following significant assumptions must hold: (1) all relevant effect modifiers must be measured and available; (2) treatment effect heterogeneity can be adequately modeled; and (3) contextual factors (e.g., healthcare system differences, practice patterns, patient case-mix) are either captured in data or sufficiently similar across populations. The strength of transportability evidence ultimately depends on the quality of the external data source and the plausibility of these assumptions. When assumptions are violated, such as when critical effect modifiers are unmeasured or healthcare system differences are substantial, then transportability analyses may yield biased results with limited generalizability. In the authors’ experience, HTA bodies have historically preferred geographically proximate (local or EU) evidence. Although this preference predates the JCA, the earlier and coordinated timing of assessments under the JCA framework may elevate the strategic importance of anticipating such expectations. Therefore it remains uncertain whether Member States will accept transportability-adjusted evidence from distant populations (e.g., US, Asian registries) as substitutes for regional RWE, particularly when the assumptions supporting such analyses are difficult to validate.

### Data Access, Governance, and Transparency

Operational feasibility under the 100-day JCA timeline is strongly shaped by data access, governance, and transparency requirements. Access to high-quality RWD may be delayed by contractual negotiations, ethics approvals, data use agreements, and data protection impact assessments. Compliance with the General Data Protection Regulation (GDPR) [9] and national privacy frameworks further complicates cross-border data pooling and registry linkage. Governance heterogeneity across Member States can therefore become as limiting as data availability itself. Therefore, early assessments of data availability, timelines for extraction/abstraction, and data governance requirements are essential. Transparency is equally critical. Pre-registration of RWE protocols and statistical analysis plans, with clear pre-specification of estimands, adjustment methods, and sensitivity analyses, strengthens credibility and enhances robustness of evidence under the compressed JCA timeframe.

## Opportunities

Despite challenges, the new JCA process offers opportunities for MAHs that plan ahead and integrate HTA and regulatory evidence generation. Cross-functional coordination between regulatory, HEOR, and clinical development teams can support dual-purpose evidence generation. For example, if seeking regulatory approval based on a single-arm trial, an external comparator arm may be used both for contextualizing the trial as well as to inform PICOs.

Beyond organizational integration, the JCA timeline creates opportunities for proactive methodological planning. In preparing for JCA submission, MAHs can proactively generate evidence for anticipated PICOs through non-interventional, fit-for-purpose study designs that integrate multiple sources of evidence [10]. For example, RWD platforms can be leveraged to identify and characterize relevant effect modifiers in advance, thereby enabling subsequent transportability analyses or population-adjusted indirect comparisons to be pre-specified rather than post-hoc, a distinction that may increase credibility within HTA review. For therapies relying on comparative effectiveness evidence from external comparators, MAHs can implement doubly-robust methods combining multiple adjustment approaches (e.g., propensity score weighting with outcome regression, or G-computation with matching) to strengthen evidence against violations of key modeling assumptions. While such advanced methods increase analytical complexity, transparent pre-specification, coupled with sensitivity analyses exploring the impact of unobserved effect modifiers, can strengthen the evidence package.

Early collaboration between clinical development, HEOR and regulatory teams is essential to ensure that methodological choices are appropriate and align with evidentiary expectations. Early multi-stakeholder engagement may help mitigate evidence misalignment prior to formal JCA timelines. Mechanisms such as joint scientific consultations with EMA and HTA bodies offer opportunities to prospectively discuss study design, comparator selection, endpoints, and the potential role of RWE. Although such advice cannot fully anticipate PICOs, it may clarify methodological expectations and reduce uncertainty. In this setting, MAHs may consider establishing a formal pre-submission RWE working group with cross-functional representation (e.g., clinical development, regulatory, epidemiology, biostatistics, and HEOR) to coordinate evidence strategy and governance. To formalize these considerations, a structured JCA readiness framework established prior to EMA notification may help anticipate evidence-related and operational constraints. Given the compressed timeline, many operational elements must be established in advance of submission. Table 1 outlines a proposed preparatory checklist that integrates PICO forecasting, RWE feasibility assessment, analytic pre-specification, transportability planning, and governance readiness. These domains reflect core sources of risk under the JCA model: uncertainty in PICO specification, data availability constraints, methodological acceptability, and operational feasibility.

**Table 1:** JCA pre-submission checklist

Domain	Key questions/objectives	Operational deliverable
PICO Scenario Forecasting	Identify plausible populations, comparators, and outcomes across major Member States; Identify heterogeneity in standards of care	Structured PICO-forecast matrix prioritizing scenarios by likelihood and strategic relevance
EU Real-World Data Landscape Assessment	Assess availability of geographically relevant RWD sources, including capture of key clinical variables and effect modifiers; Evaluate sample size adequacy	Data feasibility assessment documenting accessible datasets and variable completeness
External Comparator and Indirect Comparison Strategy	Explore suitability of existing RWD or trial data for comparative analyses; Align with anticipated endpoints	Draft statistical analysis plan templates for external comparator arms or population-adjusted indirect comparisons
Transportability Feasibility Assessment	Identify effect modifiers Assess contextual differences (e.g., care pathways, subsequent therapies; Evaluate plausibility of assumptions	Pre-specified transportability framework including sensitivity analysis plan
Data Governance and Access Planning	Establish data use agreements, ethical approvals, privacy compliance, anticipated data extraction timelines	Documented governance plan aligned with 100-day execution feasibility
Cross-Functional Evidence Coordination	Align across and clarify roles of clinical development, regulatory, epidemiology, and HEOR functions for JCA dossier preparation	Formalized cross-functional working structure or evidence steering group

## Conclusion and Call to Action

The EU JCA process, embedded into EMA review timelines, marks a major shift toward greater coordination in evidence evaluation across Member States. Although MAHs face substantial challenges such as uncertain PICOs, compressed timelines, and divergent regulatory and HTA evidentiary standards, the process also enables more rigorous, integrated evidence generation. Success will depend on early alignment across key functional teams, proactive planning of RWE and external comparator strategies, and transparent assessment around transporting evidence across geographies. Rather than framing evidence needs as "local versus external," MAHs should prioritize internally valid, fit-for-purpose evidence supplemented with clear justification of applicability to each PICO. Additionally, practical tools such as structured PICO-forecast matrices, pre-specified RWE analytic plans, JCA readiness checklists (Table 1), and early multi-stakeholder consultations (i.e., parallel regulatory–HTA scientific advice), may help operationalize this shift from reactive to proactive evidence generation. Establishing these practices will help anticipate evidence requirements, reduce late-stage uncertainty, and ensure that RWE is both robust and policy-relevant across Member States. Early cross-functional planning is critical to translating the JCA framework into timely, high-quality evidence generation supporting equitable and timely patient access to innovative, effective, and safe therapies across all 27 Member States.

## Data Availability

No datasets were generated or analysed during the current study.
